# Cell-Autonomous Regulation of Dendritic Spine Density by PirB

**DOI:** 10.1523/ENEURO.0089-16.2016

**Published:** 2016-10-07

**Authors:** George S. Vidal, Maja Djurisic, Kiana Brown, Richard W. Sapp, Carla J. Shatz

**Affiliations:** Departments of Biology and Neurobiology, and Bio-X, James H. Clark Center, Stanford University, Stanford, California 94305

**Keywords:** cell-autonomous regulation, dendritic spine density, experience-dependent plasticity, PirB, structural plasticity, synapse pruning

## Abstract

Synapse density on cortical pyramidal neurons is modulated by experience. This process is highest during developmental critical periods, when mechanisms of synaptic plasticity are fully engaged. In mouse visual cortex, the critical period for ocular dominance (OD) plasticity coincides with the developmental pruning of synapses. At this time, mice lacking paired Ig-like receptor B (PirB) have excess numbers of dendritic spines on L5 neurons; these spines persist and are thought to underlie the juvenile-like OD plasticity observed in adulthood. Here we examine whether PirB is required specifically in excitatory neurons to exert its effect on dendritic spine and synapse density during the critical period. In mice with a conditional allele of PirB (PirB^fl/fl^), PirB was deleted only from L2/3 cortical pyramidal neurons *in vivo* by timed *in utero* electroporation of Cre recombinase. Sparse mosaic expression of Cre produced neurons lacking PirB in a sea of wild-type neurons and glia. These neurons had significantly elevated dendritic spine density, as well as increased frequency of miniature EPSCs, suggesting that they receive a greater number of synaptic inputs relative to Cre^–^ neighbors. The effect of cell-specific PirB deletion on dendritic spine density was not accompanied by changes in dendritic branching complexity or axonal bouton density. Together, results imply a neuron-specific, cell-autonomous action of PirB on synaptic density in L2/3 pyramidal cells of visual cortex. Moreover, they are consistent with the idea that PirB functions normally to corepress spine density and synaptic plasticity, thereby maintaining headroom for cells to encode ongoing experience-dependent structural change throughout life.

## Visual Abstract

**Figure:**
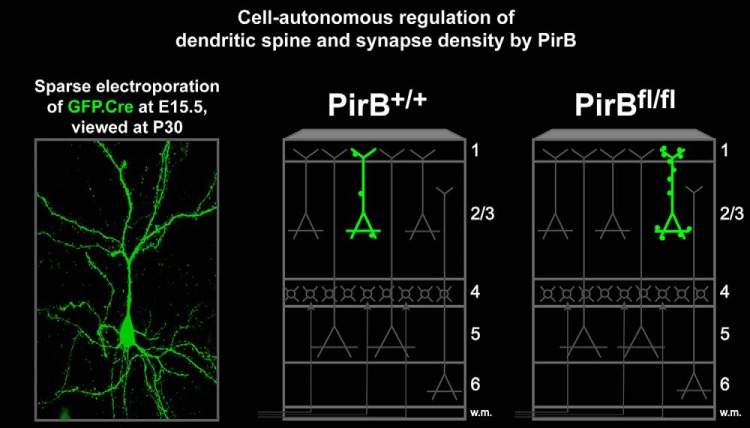

## Significance Statement

Dendritic spines, postsynaptic sites of excitatory synapses on pyramidal neurons, are regulated by experience and synaptic plasticity. Paired Ig-like receptor B (PirB) is known to restrict the extent of experience-dependent plasticity in visual cortex. Here we report that when PirB is removed *in vivo* from just a few isolated pyramidal neurons in layer 2/3 of mouse visual cortex, spine density as well as the frequency of miniature synaptic currents (a measure of the density of functional synapses) are elevated selectively in the cells lacking PirB. These results suggest that PirB expression in individual neurons is sufficient to limit excitatory synaptic density on pyramidal neurons. This cell-intrinsic function of PirB could serve to ensure that pyramidal cells have sufficient structural reserve to encode new experiences.

## Introduction

Cortical circuits are altered by experience throughout life and undergo extensive restructuring during early developmental critical periods. Underlying these experience-dependent circuit changes are cellular and molecular mechanisms of synaptic plasticity. Different learning and plasticity paradigms involving specific cortical regions result in a persistent increase in the density of dendritic spines, which are postsynaptic anatomical structures at excitatory synapses and represent sites of plasticity. This increase in dendritic spine density is thought to represent a structural trace of new learning. For example, mice trained on a forepaw reaching task show an increase in dendritic spine density on apical dendrites of L5 pyramidal cells in motor cortex (Xu et al., 2009b; Fu et al., 2012); changes are also seen in spine density on the dendrites of L2/3 pyramidal cells (Ma et al., 2016). When these newly formed spines are selectively disassembled, motor memories are erased (Hayashi-Takagi et al., 2015). In the binocular zone of mouse visual cortex, closure of one eye (monocular deprivation) generates an experience-dependent form of plasticity known as ocular dominance (OD) plasticity (Gordon and Stryker, 1996). This plasticity is accompanied by an enduring increase in spine density along the apical tufts of L5 pyramidal neurons (Hofer et al., 2009; Djurisic et al., 2013). As in motor cortex, this net increase in density is thought to provide a structural substrate that mediates a lower threshold for OD plasticity when the same eye is closed again later in life (Hofer et al., 2006, 2009).

In recent years, a list of molecules that appear to function normally as negative regulators of visual cortical plasticity has emerged, in the sense that gene knockout enhances OD plasticity following brief monocular deprivation. Blockade, removal, or genetic deletion of each of these molecules, including Nogo Receptor 1 (NgR1; McGee et al., 2005; Frantz et al., 2016), Lynx1 (Morishita et al., 2010; Bukhari et al., 2015), Death Receptor 6 (DR6; Marik et al., 2013), chondroitin sulfate proteoglycans (Pizzorusso et al., 2002), or paired Ig-like receptor B (PirB; Syken et al., 2006; Djurisic et al., 2013; Bochner et al., 2014), generates enhanced OD plasticity even in adult mice, well after normal closure of the critical period, and when significant OD plasticity cannot be elicited in wild-type mice. In addition, cell type-specific deletion of PirB from excitatory pyramidal neurons is sufficient to generate enhanced OD plasticity in adult visual cortex (Bochner et al. 2014). In mice with germline deletion (PirB^−/−^), dendritic spine density is elevated not only during the critical period but also in adults, implying a developmental pruning defect (Djurisic et al., 2013). This elevation in spine density is thought to mediate the juvenile-like OD plasticity observed in adult visual cortex of these mice.

PirB is expressed in cortical pyramidal neurons. It was discovered in an *in situ* hybridization screen designed to identify receptors expressed in brain that bind MHC class I molecules, which are involved in activity-dependent plasticity (Syken et al. 2006; Adelson et al., 2012; Djurisic et al., 2013; Bochner et al., 2014; Adelson et al., 2016). The elevated spine density and enhanced OD plasticity in visual cortex of PirB^−/−^ mice could arise from a requirement for PirB function exclusively in neurons. Microglia are also intimately involved in synapse pruning (Schafer et al., 2012; Parkhurst et al., 2013), but PirB expression has not been detected in this cell type *in vivo*. Nevertheless, studies of germline PirB knock-out mice, in which both brain and immune systems are affected, cannot distinguish between these alternatives. Here we use timed *in utero* electroporation of Cre recombinase into developing PirB^fl/fl^ mouse ventricular zone (Saito and Nakatsuji, 2001) to investigate whether neuron-specific deletion of PirB is sufficient to explain changes in dendritic spine density seen in PirB^−/−^ mice (Djurisic et al., 2013) or following pharmacological blockade of PirB in adult wild-type visual cortex (Bochner et al. 2014). In addition, *in utero* electroporation permits sparse deletion of PirB in single L2/3 neurons embedded in a wild-type environment. This type of mosaic approach has been used *in vivo* to determine whether a particular gene function is cell autonomous (Hippenmeyer et al., 2010; Akbik et al., 2013; Lu et al., 2013). Here, sparse deletion of PirB in L2/3 pyramidal neurons demonstrates that neuronal PirB is required for the regulation of synaptic density, leading us to conclude that this function of PirB is cell intrinsic.

## Materials and Methods

### Mice

**Figure 1.**
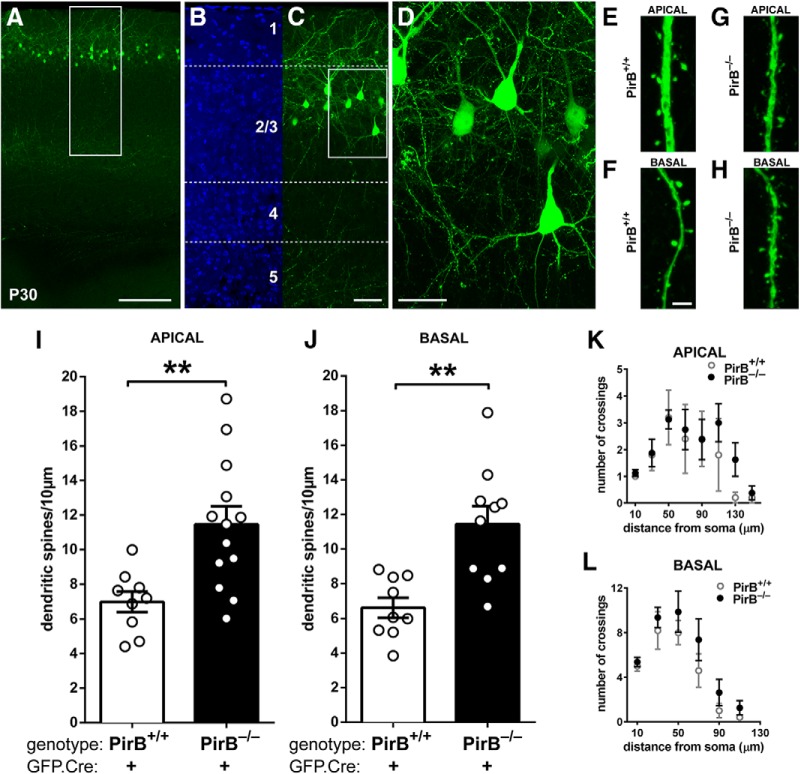
Density of dendritic spines on L2/3 pyramidal neurons is greater in visual cortex of germline PirB^−/−^ mice than in PirB^+/+^ mice at P30. ***A***, A low-magnification fluorescence micrograph of P30 mouse visual cortex showing GFP expression (green) in cells in L2/3 after GFP.Cre electroporation at E15.5. ***B***, ***C***, Higher-magnification views of boxed region shown in ***A***; DAPI nuclear counterstain (***B***) shows that most of the GFP^+^ neurons (***C***) are in layer 2 and upper layer 3. Soluble GFP fills the cells: cell bodies and dendrites, as well as descending axons clustering within layer 5 are all clearly visible. ***D***, High-magnification maximum intensity projection of boxed region shown in ***C***. Dendritic spines and axonal boutons are visible. ***E–H***, High-magnification fluorescent micrographs showing apical (***E***, ***G***) and basal (***F***, ***H***) dendritic spines in PirB^+/+^ and PirB^−/−^ mice. ***I***, Apical dendritic spine density on L2/3 pyramidal neurons of PirB^−/−^ visual cortex is elevated compared with PirB^+/+^ (PirB^+/+^ GFP^+^;Cre^+^: 7.0 ± 0.6 dendritic spines/10 μm of dendrite length, *n* = 9 cells, 5 mice; PirB^−/−^: GFP^+^;Cre^+^: 11.5 ± 1.0, *n* = 13 cells, 5 mice; *p* = 0.004^a^, one-way ANOVA with *post hoc* Bonferroni’s multiple comparisons). ***J***, Basal dendritic spine density is also increased in PirB^−/−^ mice compared with PirB^+/+^ mice (PirB^+/+^ GFP^+^;Cre^+^: 6.6 ± 0.6 dendritic spines/10 μm of dendrite length, *n* = 9 cells, 5 mice; PirB^−/−^ GFP^+^;Cre^+^: 11.4 ± 1.1, *n* = 10 cells, 5 mice; *p* = 0.002^b^, one-way ANOVA with *post hoc* Bonferroni’s multiple comparisons). ***K***, ***L***, Sholl analysis reveals no significant changes in apical (***K***) or basal (***L***) dendritic branching between PirB^+/+^ and PirB^−/−^ L2/3 neurons (PirB^+/+^ GFP^+^;Cre^+^: *n* = 5 cells, 3 mice; PirB^−/−^ GFP^+^;Cre^+^: *n* = 8 cells, 5 mice; *p* = 0.2443^c^ (***K***), *p* = 0.0574^d^ (***L***), two-way ANOVA with repeated measures). **p < 0.01. Scale bars: ***A***, 0.2 mm; ***B***, ***C***, 50 μm; ***D***, 25 μm; ***E–H***, 3 μm.

PirB^fl/fl^, PirB^+/+^, and PirB^−/−^ mice were generated by Syken et al. (2006). Briefly, PirB^fl/fl^ mice were generated by electroporating a construct with loxP sites flanking exons 10–13 of PirB into the 129 J1 ES line derived from agouti 129S4/SvJae mice. Exons 10–13 of PirB code for the transmembrane domain of PirB, as well as part of the intracellular domain encompassing the signaling immunoreceptor tyrosine-based inhibitory motifs. Thus, Cre-mediated excision of exons 10–13 from the PirB gene in PirB^fl/fl^ mice results in a truncated protein that is unable to signal, as shown previously by anti-phosphotyrosine immunoprecipitation experiments (Syken et al., 2006). To generate mice with germ line deletion of PirB (i.e., PirB^−/−^), a deleter strain that targets Cre-recombinase expression to early mouse embryo via adenovirus EIIa promoter (B6.FVB-TGN(EIIa-cre)C5379Lmgd; The Jackson Laboratory) was crossed to PirB^fl/fl^ mice. Heterozygote sibling matings were then used to generate both control PirB^+/+^ line and a homozygous PirB^−/−^ line (Syken et al., 2006). PirB^fl/fl^, PirB^+/+^, and PirB^−/−^ mice were maintained as three separate lines on the same mixed genetic background (C57BL/6 × SV/129J). Previous studies have shown that the excision of PirB from PirB^fl/fl^ by Cre recombinase under control of the UbC promoter occurs within 1 week (Bochner et al., 2014), and is accompanied by a complete loss of PirB protein after ∼3 weeks from the onset of Cre recombinase expression (Fig. 1; Bochner et al., 2014). Cre recombinase expression via the GFP.Cre construct under the phosphoglycerate kinase promoter used here for the electroporation experiments described below should be even more rapid and efficient (Qin et al., 2010).

All experiments were performed in accordance with the *Guide for the Care and Use of Laboratory Animals* of the National Institutes of Health and approved by the Stanford University Institutional Animal Care and Use Committee. Experimental methods are also in accordance with the policies of the Society for Neuroscience on the Use of Animals and Humans in Neuroscience Research. All mice were maintained in a pathogen-free environment.

### *In utero* electroporation

Female mice were mated within the same line (PirB^+/+^, PirB^fl/fl^, or PirB^−/−^) and checked daily for vaginal plugs. The day that a plug was found was counted as embryonic day 0.5 (E0.5). *In utero* electroporation was performed at E15.5, when L2/3 cortical neurons are generated (Saito and Nakatsuji, 2001; Tabata and Nakajima, 2001; Saito, 2006; Chen et al., 2008). Pregnant mice were anesthetized using 1–2.5% isoflurane in 100% O_2_. Using a sterile surgical technique, a midline incision was made to the abdominal wall to expose the uterine horns. Lateral ventricles of embryos were injected with either 1.0 or 1.5 μl of 1.9–2.0 μg/μl GFP.Cre in Tris-EDTA buffer (10 mm Tris-HCl, pH 8.0, and 1 mm ethylenediaminetetraacetic acid); injection of the lower (1.0 μl) volume of DNA was critical to achieving sparse electroporations.

GFP.Cre is an expression construct in which GFP expression is driven by the ubiquitin C promoter, and Cre expression is driven separately by the phosphoglycerate kinase promoter (gift from Tyler Jacks, Koch Institute for Integrative Cancer Research at the Massachusetts Institute of Technology, Cambridge, Massachusetts; plasmid #20781, Addgene; Andreu-Agullo et al., 2011; Scotto-Lomassese et al., 2011). Injections were achieved using micropipettes made from glass capillary tubes (TW100F-4, World Precision Instruments) pulled into a fine tip with a micropipette puller (P-97, Sutter Instruments). Tweezer-type circular electrodes (5 mm in diameter) were custom made by coiling 24 AWG platinum wire (PTP201, World Precision Instruments) and were used to deliver five 50 ms electric pulses at 45 V with 950 ms intervals, using a square-wave generator (ECM 830, BTX). The exposed uterus was kept moist with 0.9% saline at 37°C. After each electroporation procedure, dams were given buprenorphine intraperitoneally (0.1 mg/kg; catalog #2808, Tocris Bioscience), the abdominal wall was sutured shut, and the dam was allowed to recover in a cage kept at ∼37^°^C. Pregnant dams were monitored postoperatively until after giving birth and able to nurse pups.

#### Histology

Mice received an overdose of sodium pentobarbital (>86 mg/kg) and sodium phenytoin cocktail (Beuthanasia-D, Merck; >11 mg/kg, i.p.), and brain tissue was fixed via transcardial perfusion of ice-cold 0.1 m sodium PBS followed by ice-cold 4% (w/v) paraformaldehyde in 0.1 m PBS. Brains were then postfixed overnight in 4% (w/v) paraformaldehyde in PBS at 4ºC, followed by 12–24 h in PBS at 4ºC. Brains were cut coronally using a vibrating microtome (VT1200S, Leica Microsystems) into 150-μm-thick sections. Sections were mounted on Superfrost Plus glass slides (VWR) with ProLong Gold antifade reagent (Invitrogen) as a mounting medium and covered with #1.5 thickness cover glass (VWR).

#### Dendritic spine and axonal bouton imaging and analysis

Slides of histological sections prepared as described above were initially screened for GFP fluorescence using low-powered objectives on an Eclipse E800 microscope (Nikon), without knowledge of genotype. GFP^+^ neurons in L2/3 of visual cortex were then identified by comparing landmarks observed via DAPI fluorescence and bright-field imaging (Eclipse E800 microscope), including the shape of the internal capsule, the hippocampus, thalamic structures, and cytoarchitectonic differences between the cortical layers (Paxinos and Franklin, 2008). Labeled cells with low expression levels of GFP and without complete primary dendritic arbors were excluded from further imaging. High-resolution images of apical and basal dendrites of L2/3 pyramidal neurons in visual cortex, and of continuous 100–300 μm segments of descending axons in L5, were taken on an SP2 or SP8 confocal microscope (Leica Microsystems), or on a two-photon microscope (Prairie Technologies); 63×/1.40 numerical aperture (NA) oil-immersion (Leica) or 60×/1.1 NA water-immersion (Prairie) objectives were used. Images were acquired at or over the theoretical Nyquist sampling rate for each objective used (∼71 nm/pixel). All dendritic spine and axonal bouton analysis was performed manually while blind to genotype using ImageJ software (National Institutes of Health); manual tracing with ImageJ Simple Neurite Tracer plugin was used for length measurement of neurites. Axonal boutons were identified as in De Paola et al. (2006).

#### Sholl analysis

Images acquired by two-photon microscopy using 10× or 40× objectives (Prairie) that contained the entire L2/3 neuron dendritic arbor in a 150-µm-thick section were used for Sholl analysis (Sholl, 1953). For each cell, concentric circles were drawn at 10, 30, 50, 70, 90, 110, and 130 μm from the geometric center of the soma (0 μm), using the Concentric Circles ImageJ plugin. The number of crossings of dendritic branches belonging to individual cells through each circle were counted, and used as the measure of the complexity of the dendritic arbor.

#### Assessment of neuronal labeling density resulting from electroporations

**Figure 2.**
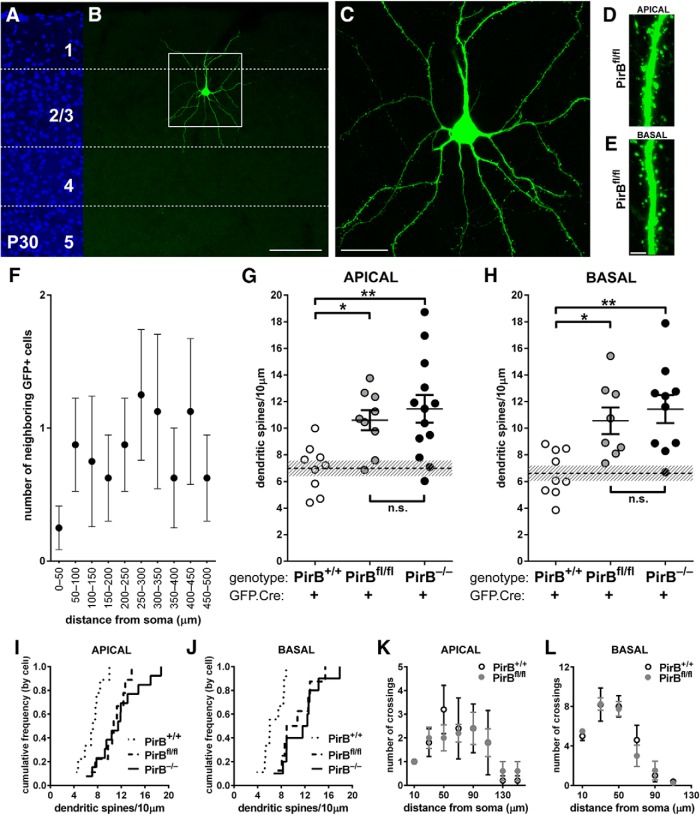
Dendritic spine density at P30 is elevated on isolated PirB^−/−^ neurons in layer 2/3 after sparse excision of PirB at E15.5. ***A***, ***B***, Fluorescent micrographs at P30 of nuclear counterstain (DAPI; ***A***) and an isolated L2/3 pyramidal neuron electroporated with GFP.Cre (***B***) from a visual cortex section from a P30 PirB^fl/fl^ mouse. ***C***, High-magnification maximum intensity projection of the boxed area in ***B***. ***D***, ***E***, Zoomed-in high-magnification micrographs of portions of apical (***D***) and basal (***E***) dendrites showing dendritic spines in PirB^fl/fl^ mice. ***F***, Number of neighboring GFP^+^;Cre^+^ cells as a function of distance from a neuron of interest in PirB^fl/fl^ tissue (*n* = 8 cells, 7 mice). The graph shows that, on average, there was only one GFP^+^;Cre^+^ cell in every 50 μm increment analyzed. ***G***, Spine density on apical dendrites is greater in GFP^+^;Cre^+^ neurons from PirB^fl/fl^ mice than PirB^+/+^ mice (PirB^+/+^: 7.0 ± 0.6 dendritic spines/10 μm of dendritic length, *n* = 9 cells, 5 mice; same data as in Fig. 1*I*; PirB^fl/fl^, GFP^+^;Cre^+^: 10.6 ± 0.8, *n* = 9 cells, 7 mice; PirB^−/−^, GFP^+^;Cre^+^: 11.5 ± 1.0, *n* = 13 cells, 5 mice; same data as in Fig. 1*I*; PirB^+/+^ vs PirB^fl/fl^, *p* = 0.041^a^; PirB^fl/fl^ vs PirB^−/−^, *p* = 1.000^a^, one-way ANOVA with *post hoc* Bonferroni’s multiple comparisons). ***H***, Basal dendritic spine density is greater in GFP^+^; Cre^+^ neurons from PirB^fl/fl^ mice than PirB^+/+^ mice (PirB^+/+^: 6.6 ± 0.6 dendritic spines/10 μm of dendritic length, *n* = 9 cells, 5 mice; data from Fig. 1*J*; PirB^fl/fl^, GFP^+^;Cre^+^: 10.6 ± 1.0, *n* = 8 cells, 7 mice; PirB^−/−^, GFP^+^;Cre^+^: 11.4 ± 1.1, *n* = 10 cells, 5 mice; data from Fig. 1*J*; PirB^+/+^ vs PirB^fl/fl^ *p* = 0.02^b^, PirB^fl/fl^ vs PirB^−/−^ *p* = 1.000^b^, One-way ANOVA with *post hoc* Bonferroni's multiple comparisons). ***I***, Cumulative histogram (by cell) of data presented in ***G***. ***J***, Cumulative histogram (by cell) of data presented in ***H***. ***K***, ***L***, Sholl analysis reveals no significant changes in apical (***K***) or basal (***L***) dendritic branching between GFP^+^;Cre^+^ L2/3 neurons from PirB^+/+^ and PirB^fl/fl^ mice (***K***: PirB^+/+^, *n* = 5 cells, 3 mice; data from Fig. 1*K*; PirB^fl/fl^, *n* = 5 cells, 3 mice, *p* = 0.8864^e^; ***L***: PirB^+/+^, *n* = 5 cells, 3 mice; data from Fig. 1*L*; PirB^fl/fl^, *n* = 4 cells, 3 mice, *p* = 0.7799^f^, two-way ANOVA with repeated measures). *p < 0.05; **p < 0.01. Scale bars: ***A***, ***B***, 100 μm; ***C***, 25 μm; ***D***, ***E***, 3 μm.

Low-magnification images of L2/3 GFP^+^;Cre^+^ PirB^fl/fl^ neurons were acquired (Eclipse E800 microscope) that included all GFP^+^ neurons within a 0.5 mm radius of a GFP^+^ neuron of interest. For each cell, concentric circles were drawn at 50 μm intervals from the geometric center of the soma (0 μm), using the Concentric Circles ImageJ plugin. The number of GFP^+^ cells located within each ring (e.g., 50–100 μm) was then counted to derive a density versus distance measurement of distribution of the electroporated neighboring cells surrounding a neuron of interest (Fig. 2*F*).

#### Electrophysiology

At postnatal day 28 (P28) to P32, PirB^+/+^ and PirB^fl/fl^ mice that had been electroporated *in utero* at E15.5 were overdosed with an intraperitoneal injection of a ketamine (132 mg/kg; Phoenix), xylazine (14 mg/kg; Akorn), acepromazine (0.2 mg/kg; Boehringer Ingelheim) cocktail; after deep anesthesia, mice were decapitated. Brains were immediately removed and immersed in *N*-methyl-d-glucamine (NMDG)-based ice-cold slicing buffer, to minimize damage from excitotoxicity and hypoxia (composition: 135 mm NMDG, 1 mm KCl, 1.2 mm KH_2_PO_4_, 1.5 mm MgCl_2_, 0.5 mm CaCl_2_, 20 mm choline bicarbonate, and 10 mm glucose, pH 7.4, equilibrated with 95% O_2_ and 5% CO_2_). Brains were cut coronally at 400 μm, using a vibrating microtome (VT1000S, Leica Microsystems). Sections were transferred to a recovery chamber containing high-magnesium artificial CSF (ACSF; composition: 125 mm NaCl, 26 mm NaHCO_3_, 2.3 mm KCl, 1.26 mm KH_2_PO_4_, 4.0 mm MgCl_2_, 2.5 mm CaCl_2_, and 20 mm glucose, pH 7.4, equilibrated with 95% O_2_ and 5% CO_2_). Slices recovered at 37°C for 30 min, and an additional 30 min at room temperature before recording (Crozier et al., 2007; Djurisic et al., 2013).

The whole-cell patch-clamp technique was used to record miniature EPSCs (mEPSCs) from individual pyramidal cells in slices containing primary visual cortex and binocular zone. Single, GFP-labeled or unlabeled L2/3 pyramidal neurons in visual cortex were visualized using infrared differential interference contrast illumination combined with fluorescent illumination in an Olympus BX51WI Microscope via a 60× water-immersion objective coupled with an additional 2× zoom lens (120× final magnification). For GFP^−^ cells, only those with pyramidal-shaped somata and a visible apical dendrite oriented perpendicular to the pial surface were selected for recordings. The external bath solution (ACSF) was maintained at ∼30°C, and bubbled with 95% O_2/_5% CO_2_ for the duration of the experiment. Patch-clamp electrodes (1.5–4.2 MΩ) were filled with cesium-based intracellular solution (composition: 135 mm CsCl, 10 mm HEPES, 1 mm EGTA, 4 mm Mg-ATP, 0.4 mm Na-GTP, pH 7.4 with NaOH). To reveal the morphology of neurons from which mEPSCs were recorded, biocytin (catalog #B4261, Sigma-Aldrich) was added to the intracellular solution at a final concentration of 5 mg/ml; after recording, sections were fixed in 4% paraformaldehyde overnight, followed by permeabilization and staining with Texas Red-conjugated avidin (catalog #A-2006, Vector Laboratories; 1:10). Once in whole-cell mode, the pipette solution equilibrated with the cell contents for 5–10 min before recording. Miniature excitatory postsynaptic AMPA currents were isolated pharmacologically using 1 μm tetrodotoxin (catalog #1078, Tocris Bioscience), 50 μm aminophosphonopentanoic acid (catalog #A5282, Sigma-Aldrich), and 10 μm gabazine (SR95531; catalog #1262, Tocris Bioscience). Series resistance was 9–20 MΩ. The low-noise AxoPatch 200B Patch-Clamp Amplifier, DigiData 1322A Digitizer, and Clampex Software (Molecular Devices) were used for data collection. Before analysis, recordings across all conditions were filtered with a 60 Hz, three-harmonic, 100 cycle electrical interference filter (Molecular Devices), as well as a Gaussian, 53 coefficient low-pass filter (setting, −3 dB to 2 kHz; Molecular Devices). Analysis of mEPSCs was performed using MiniAnalysis version 6.0.7 (Synaptosoft), and blinded to both genotype and cell identity (i.e., GFP^+^ vs GFP^–^). Cells with cell capacitance of 60 pF and/or leak current more negative than −150 pA were excluded from the analysis. Root mean square (rms) baseline noise was calculated from three different places during the 10 minute recording, and was <3 pA. Automatic detection parameters in MiniAnalysis were adapted from the studies of Xu et al. (2009a) and Muhia et al. (2012), and were as follows: threshold was set to 4 times the rms noise value; the period to search for a local maximum was 20 ms; the time before peak to establish baseline was 5 ms; the period to search a decay time was 5 ms; the fraction of the peak to find a decay time was 0.5; the period to average baseline was 2 ms; the area threshold was five times the rms noise value; and the number of points to average to establish the peak was 3. Continuous automatic analysis was run during 1–2 min of the 10 min recording in which the leak current was most stable; detected events were manually inspected to eliminate false positives.

### Statistics

All statistical analyses and graphs were performed with GraphPad Prism software, and power analyses were performed using G*Power software (Heinrich-Heine-Universität Düsseldorf, Düsseldorf, Germany; http://www.gpower.hhu.de/ ; Faul et al., 2007, 2009). Data are presented as the mean ± SEM; sample size (*n*) is the number of cells analyzed, followed by the number of mice used for the entire sample, unless noted otherwise in the text (Table 1). Analyses were performed blinded to genotype and condition.

**Table 1:** Statistical table

	Data structure	Type of test	Power (α = 0.05)	*p* value
a (Figs. 1I, 2G)	Normality assumed (Levene’s test *p* = 0.118)	One-way ANOVA with Bonferroni’s multiple-comparisons *post hoc*	0.9403	PirB^+/+^ Cre^+^ vs PirB^−/−^ Cre^+^, *p* = 0.004; PirB^+/+^ Cre^+^ vs PirB^fl/fl^ Cre^+^, *p* = 0.041; PirB^fl/fl^ Cre^+^ vs PirB^−/−^ Cre^+^, *p* = 1.000
b (Figs. 1J and 2H)	Normality assumed (Levene’s test *p* = 0.233)	One-way ANOVA with Bonferroni’s multiple-comparisons *post hoc*	0.9733	PirB^+/+^ Cre^+^ vs PirB^−/−^ Cre^+^, *p* = 0.002; PirB^+/+^ Cre^+^ vs PirB^fl/fl^ Cre^+^, *p* = 0.02; PirB^fl/fl^ Cre^+^ vs PirB^−/−^ Cre^+^, *p* = 1.000
c (Fig. 1K)	Normal distribution	Two-way ANOVA with repeated measures	0.2383	0.2443
d (Fig. 1L)	Normal distribution	Two-way ANOVA with repeated measures	0.9534	0.0574
e (Fig. 2K)	Normal distribution	Two-way ANOVA with repeated measures	0.0501	0.8864
f (Fig. 2L)	Normal distribution	Two-way ANOVA with repeated measures	0.0521	0.7799
g (Fig. 3C)	Normality assumed (Levene’s test *p* = 0.494)	One-way ANOVA with Bonferroni’s multiple-comparisons *post hoc*	0.9988	PirB^+/+^ Cre^+^ vs PirB^fl/fl^ Cre^+^, *p* = 0.924; PirB^+/+^ Cre^+^ vs PirB^−/−^ Cre^+^, *p* = 1.000; PirB^fl/fl^ Cre^+^ vs PirB^−/−^ Cre^+^, *p* = 1.000; P23 PirB^+/+^ vs P30 PirB^+/+^, *p* = 0.004; P23 PirB^fl/fl^ vs P30 PirB^fl/fl^, *p* = 0.19; P23 PirB^−/−^ vs P30 PirB^−/−^, *p* = 1.00
h (Fig. 4G)	Normality not assumed	Mann–Whitney	0.8509	0.0058
i (Fig. 4I)	Normality not assumed	Mann–Whitney	0.1634	0.3286
j (Fig. 4K)	Normality not assumed	Mann–Whitney	0.0961	0.4940
k (Fig. 4M)	Normality not assumed	Mann–Whitney	0.1855	0.3918
l (Fig. 5E)	Normality not assumed	Wilcoxon signed-rank	0.9959	0.0060
m (Fig. 5F)	Normality not assumed	Wilcoxon signed-rank	0.1109	0.7722
n (Fig. 6C)	Normality assumed (Levene’s test *p* = 0.869)	One-Way ANOVA with Bonferroni’s multiple comparisons *post hoc*	0.1347	PirB^+/+^ Cre^+^ vs PirB^fl/fl^ Cre^+^, *p* = 1.000; PirB^+/+^ Cre^+^ vs PirB^−/−^ Cre^+^, *p* = 1.000; PirB^fl/fl^ Cre^+^ vs PirB^−/−^ Cre^+^, *p* = 1.000

## Results

To examine whether PirB expression, specifically in pyramidal neurons, is sufficient to regulate spine density, PirB^+/+^, PirB^−/−^, and PirB^fl/fl^ mice were studied in combination with *in utero* electroporation of a GFP.Cre expression vector to selectively target a subset of excitatory pyramidal neurons in L2/3 at the time of their genesis. Because excitatory neurons from different cortical layers are generated sequentially in the cortical ventricular zone (McConnell and Kaznowski, 1991; Chen et al., 2008; Greig et al., 2013), neurons of each cortical layer can be targeted by timing *in utero* electroporations of the ventricular zone; at E15.5, L2/3 pyramidal neurons are targeted exclusively (Saito and Nakatsuji, 2001). Glia and interneurons are not transfected since the production of glial cells peaks around birth, while interneurons are generated in the ganglionic eminence and not in the ventricular zone (Anderson et al., 1997; Nery et al., 2002). Layer 2/3 pyramidal neurons are of particular interest because of the changes previously observed in PirB^−/−^ mice in cellular mechanisms of synaptic plasticity at L4 to L2/3 synapses, in mEPSC frequency recorded from L2/3 neurons, and in OD plasticity as assessed in L2/3 using intrinsic signal imaging (Djurisic et al., 2013).

### L2/3 pyramidal neurons of PirB^−/−^ mice have elevated dendritic spine density

This first experiment was designed to examine whether spine density on L2/3 neurons in PirB^−/−^ mice is increased at P30. At E15.5, PirB^+/+^ or PirB^−/−^ embryos were injected with a DNA construct (GFP.Cre) expressing GFP, as well as Cre recombinase under a separate promoter and subjected to electroporation. Note that this initial experiment was not performed in mice carrying a conditional allele of PirB. Consequently, neurons are labeled with GFP to permit the assessment of morphology, but Cre expression does not drive excision, and instead serves as a control for subsequent experiments.

Electroporation at E15.5 results in laminar-specific GFP labeling of L2/3 pyramidal neurons in primary visual cortex at P30 (Fig. 1*A–D*). Dendritic spines were extensively GFP^+^ labeled as viewed using confocal microscopy (Fig. 1*E–H*), permitting assessment of density. Spine density on L2/3 pyramidal neurons of PirB^−/−^ mice is significantly elevated, relative to that in PirB^+/+^ mice; spine density is 64% greater on apical dendrites in PirB^−/−^ versus PirB^+/+^ (Fig. 1*I*), and 73% greater on basal dendrites (Fig. 1*J*). The excess dendritic spines are likely to be sites of functional synapses, since EM studies from mouse visual cortex show that >95% of dendritic spines have machinery needed for synaptic transmission: they are positive for PSD-95 and apposed by presynaptic active zones (Arellano et al., 2007). Moreover, the spine density increase observed here in PirB^−/−^ mouse visual cortex is consistent with the previously reported increase in mEPSC frequency recorded from L2/3 pyramidal neurons—a sensitive measure of the density of functional excitatory synapses (Djurisic et al., 2013).

These significant changes in dendritic spine density in PirB^−/−^ neurons suggest that other aspects of neuronal morphology may also be altered. To examine this possibility, the dendritic complexity of GFP^+^ L2/3 pyramidal neurons in visual cortex of PirB^+/+^ and PirB^−/−^ mice was assessed using Sholl analysis (Sholl, 1953). Apical and basal dendritic complexity was unaltered in PirB^−/−^ mice relative to PirB^+/+^ mice (Fig. 1*K*,*L*). Together, these observations demonstrate that the changes in mEPSC frequency observed previously (Djurisic et al. 2013) are accompanied by changes in spine density but not dendritic complexity, in L2/3 pyramidal neurons.

### Sparse deletion in PirB^fl/fl^ mice results in elevated spine density on isolated L2/3 pyramidal neurons

An increase in both spine density (Fig. 1) and in functional excitatory synapses (Djurisic et al., 2013) is observed in L2/3 pyramidal neurons of PirB^−/−^ versus PirB^+/+^ mice. To examine whether this increase is due to the loss of PirB from the very cells where dendritic spines were counted or to the loss of PirB in other presynaptic or non-neuronal cells, a mosaic approach was used to delete PirB from isolated L2/3 pyramidal neurons. PirB^fl/fl^ mice were electroporated with GFP.Cre at E15.5, and then studied at P30 (Fig. 2*A–E*). In PirB^fl/fl^ mice, cells expressing GFP coexpress Cre recombinase, which drives the excision of the floxed PirB alleles.

In a subset of PirB^fl/fl^ mice electroporated with the GFP.Cre vector (see Materials and Methods), a very sparse distribution of PirB knock-out neurons embedded in a “sea” of cells containing intact PirB alleles was obtained (Fig. 2*A–E*). To quantify the sparseness of PirB deletion, the density of GFP^+^ cells in V1 was measured by counting the number of labeled cells in a series of concentric circles surrounding the cell selected for spine analysis (Fig. 2*F*). Using this Sholl analysis, we determined that for the majority of neurons analyzed, there were almost no neighboring GFP^+^ neurons located within a radius of 50 μm, and then within each 50 μm increment outward from the cell under analysis, there was an average of only one additional labeled neuron at most. In contrast, the density of labeled cells was far greater in Figure 1, where a larger volume of DNA was injected. Thus, electroporation could result in a very sparse deletion of PirB from isolated L2/3 pyramidal neurons, permitting us to examine a cell-intrinsic function for PirB in regulating dendritic spine density.

At P30, spine density on isolated GFP^+^;Cre^+^ L2/3 pyramidal neurons in visual cortex of sparsely electroporated PirB^fl/fl^ mice was significantly elevated, compared with GFP^+^;Cre^+^ neurons from control PirB^+/+^ mice, in which PirB excision has not occurred. In addition, density was almost identical to that observed on L2/3 dendrites of GFP^+^;Cre^+^ neurons from PirB^−/−^ mice, as shown in Figure 2*G–J*, which compares data from all three genotypes. There are significant spine density increases along both apical (52%) and basal (60%) dendrites in PirB^fl/fl^ neurons that have undergone recombination and lack PirB, compared with L2/3 GFP^+^; Cre^+^ neurons in PirB^+/+^ mice that have not undergone recombination and express PirB normally (Fig. 2*G*,*H*). Multiple comparisons with one-way ANOVA followed by Bonferroni *post hoc* test reveals significant differences between the density of spines on apical dendrites between PirB^+/+^ versus PirB^fl/fl^ mice (*p* = 0.041) and PirB^+/+^ versus PirB^−/−^ mice (*p* = 0.004), but not PirB^fl/fl^ versus PirB^−/−^ mice (*p* = 1.00). Similar statistically significant differences for basal dendritic spines were revealed by *post hoc* analysis, as follows: PirB^+/+^ versus PirB^fl/fl^ mice (*p* = 0.02) and PirB^+/+^ versus PirB^−/−^ mice (*p* = 0.002), but not PirB^fl/fl^ versus PirB^−/−^ mice (*p* = 1.00).

The dendritic arborization of GFP^+^;Cre^+^ cells in PirB^fl/fl^ mice versus GFP^+^; Cre^+^ cells in PirB^+/+^ mice was also examined. No significant differences in apical or basal dendritic Sholl profiles (Sholl, 1953) were observed (Fig. 2*K*,*L*), underscoring a selective effect of PirB deletion on spine density in isolated L2/3 neurons.

### Dendritic spine density on L2/3 pyramidal cells in all genotypes is similar at P23

The elevated spine density observed at P30 in mice lacking PirB could arise from changes in dendritic spine formation and/or from a failure of pruning. To distinguish these possibilities, the sparse GFP.Cre electroporation experiment described above was repeated, but the density of spines was assessed 1 week earlier at P23, during the period of synaptic pruning and when dendritic spine density in WT visual cortex is highest based on previous Golgi cell and EM studies (Ruiz-Marcos and Valverde, 1969; Markus and Petit, 1987).

**Figure 3.**
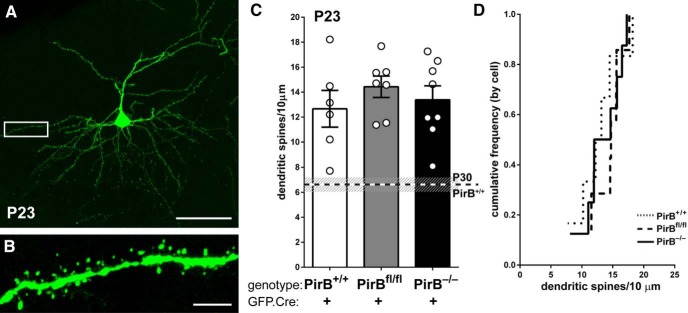
At P23, dendritic spine density on L2/3 pyramidal cells in PirB^−/−^ visual cortex is similar to that of PirB^+/+^. ***A***, Fluorescent micrograph at P23, an age near the onset of spine pruning, showing an isolated L2/3 pyramidal neuron electroporated with GFP.Cre at E15.5. ***B***, High-magnification, maximum-intensity projection of boxed area in ***A*** showing spines on basal dendrites of GFP^+^;Cre^+^ L2/3 neuron. ***C***, Basal dendrite spine density is higher in PirB^+/+^ neurons at P23 than in PirB^+/+^ neurons at P30 (P30 data indicated by dotted line; data from Fig. 2*H*); spine density from P23 PirB^+/+^ is not different from PirB^fl/fl^ or PirB^−/−^ neurons (P23 PirB^+/+^: 12.66 ± 1.5, *n* = 6 cells, 3 mice; P23 PirB^fl/fl^: 14.43 ± 0.9, *n* = 7 cells, 5 mice; P23 PirB^−/−^: 13.39 ± 1.1, *n* = 8 cells, 4 mice; P23 PirB^+/+^vs P23 PirB^−/−^, *p* = 1.000^g^; P23 PirB^+/+^ vs P23 PirB^fl/fl^, *p* = 0.924^g^; P23 PirB^fl/fl^ vs PirB^−/−^, *p* = 1.000^g^, one-way ANOVA with *post hoc* Bonferroni’s multiple comparisons; P23 PirB^+/+^ vs P30 PirB^+/+^, *p* = 0.004^g^; P23 PirB^fl/fl^ vs P30 PirB^fl/fl^, *p* = 0.19^g^; P23 PirB^−/−^ vs P30 PirB^−/−^, *p* = 1.00^g^, one-way ANOVA with *post hoc* Bonferroni’s multiple comparisons). ***D***, Cumulative histogram (by cell) of data presented in ***C***. Scale bars: ***A***, 50 μm; ***B***, 5 μm.

The dendrites and spines of L2/3 pyramidal neurons at P23 were extensively GFP labeled (Fig. 3*A*,*B*) subsequent to electroporation at E15.5. Multiple comparisons with one-way ANOVA and Bonferroni *post hoc* test did not reveal any significant differences between the density of spines for all three genotypes (Fig. 3*C*,*D*). Moreover, dendritic spine density for PirB^+/+^ is almost twice as high at P23 (Fig. 3*C*: ∼13 spines/10 μm) than at P30 (∼7 spines/10 μm). In contrast to wild-type, there are no statistically significant differences between PirB^fl/fl^ mice at P23 versus P30, or PirB^−/−^ mice at P23 versus P30, strongly suggesting that spine pruning is deficient in L2/3 neurons lacking PirB.

### Sparse PirB excision results in increased mEPSC frequency recorded from isolated L2/3 pyramidal neurons at P30

**Figure 4.**
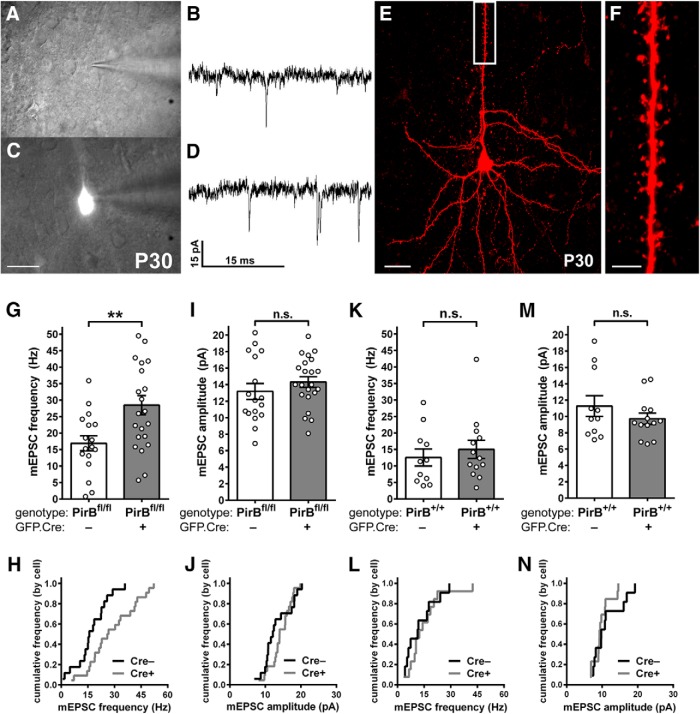
Sparse excision of PirB from PirB^fl/fl^ at E15.5 increases mEPSC frequency but not amplitude in L2/3 pyramidal neurons in P30 visual cortex. ***A***, ***C***, Combined differential interference contrast and fluorescence micrographs of neurons in visual cortex used for whole-cell recordings of mEPSCs, showing GFP**^–^**;Cre^−^ cells (***A***) and an isolated GFP^+^;Cre^+^ cell (***C***). ***B***, ***D***, Example mEPSC traces from GFP^−^;Cre^−^ (***B***) or GFP^+^;Cre^+^ (***D***) L2/3 pyramidal neurons from primary visual cortex slices of P30 PirB^fl/fl^ mice. ***E***, Fluorescent micrograph showing an L2/3 pyramidal neuron filled with biocytin during mEPSC recording, and visualized with Texas Red-conjugated avidin. ***F***, Zoomed-in, maximum-intensity projection of the boxed area in ***E***, showing apical dendritic spines. ***G***, In PirB^fl/fl^ mice, mEPSC frequency is increased in GFP^+^;Cre^+^ cells compared with GFP^−^;Cre^−^ cells (GFP^−^;Cre^−^: 16.91 ± 2.28 Hz, *n* = 17 cells, 14 slices, 8 mice; GFP^+^;Cre^+^: 28.51 ± 2.89 Hz, *n* = 22 cells, 14 slices, 8 mice; *p* = 0.0058^h^, Mann–Whitney test). ***H***, Cumulative histogram (by cell) of data presented in ***G***. ***I***, mEPSC amplitude does not differ between GFP^−^;Cre^−^ and GFP^+^;Cre^+^ cells in PirB^fl/fl^ mice (GFP^−^;Cre^−^: 13.18 ± 0.97 pA, *n* = 17, cells, 14 slices, 8 mice; GFP^+^;Cre^+^: 14.32 ± 0.64 pA, *n* = 22 cells, 14 slices, 8 mice; *p* = 0.3286^i^, Mann–Whitney test). ***J***, Cumulative histogram (by cell) of data presented in ***I***. ***K***, In control PirB^+/+^ mice, electroporation of GFP.Cre did not result in an increase in mEPSC frequency in GFP^+^;Cre^+^ relative to GFP^−^;Cre^−^, as expected (GFP^−^;Cre^−^: 12.53 ± 2.55 Hz, *n* = 11 cells, 11 slices, 6 mice; GFP^+^;Cre^+^: 15.01 ± 2.76, *n* = 13 cells, 11 slices, 6 mice; *p* = 0.4940^j^, Mann–Whitney test). ***L***, Cumulative histogram of data presented in ***K***. ***M***, In control PirB^+/+^ mice, mEPSC amplitudes are not different between GFP^+^;Cre^+^ and GFP^−^;Cre^−^ cells (GFP^−^;Cre^−^: 11.28 ± 1.26 pA, *n* = 11 cells, 11 slices, 6 mice; GFP^+^;Cre^+^: 9.73 ± 0.69, *n* = 13 cells, 11 slices, 6 mice; *p* = 0.3918^k^, Mann–Whitney test). ***N***, Cumulative histogram of data (by cell) presented in ***M***. Scale bars: ***A***, ***C***, 25 μm; ***E***, 20 μm; ***F***, 5 μm. **p < 0.01.

In PirB^−/−^ mice, the increase in spine density on L2/3 pyramidal neurons (Fig. 1) is accompanied by an increase in mEPSC frequency, a measure of the density of functional excitatory synapses (Djurisic et al., 2013). Is the increase in dendritic spine density observed following sparse deletion in PirB^fl/fl^ mice similarly accompanied by an increase in mEPSC frequency? To assess the strength and number of functional excitatory synaptic inputs, the frequency and amplitude of mEPSCs in acute slices from visual cortex of PirB^fl/fl^ mice was assessed using the patch-clamp technique (Fig. 4). In this experiment, both labeled (GFP^+^;Cre^+^) and unlabeled (GFP^−^;Cre^−^) neurons could be recorded and compared in the same slice (Fig. 4*A–D*); cells were also labeled via patch pipette with biocytin to confirm the pyramidal identity of GFP^−^;Cre^−^ cells targeted for recordings (Fig. 4*E*,*F*). The frequency of mEPSCs in GFP^+^;Cre^+^ neurons was 69% higher than in GFP^−^;Cre^−^ controls (Fig. 4*G*,*H*), with no measurable change in mEPSC amplitude (Fig. 4*I*,*J*).

Miniature EPSC frequencies and amplitudes recorded from GFP^+^;Cre^+^ neurons in PirB^fl/fl^ mice (Fig. 4*G–J*) were similar to those of L2/3 pyramidal neurons in germline PirB^−/−^ slices (Djurisic et al., 2013). In addition, GFP^−^;Cre^−^ neurons in PirB^fl/fl^ slices had mEPSC frequency and amplitudes similar to PirB^+/+^ slices (Fig. 4, compare *G–J*, *K–N*). These results indicate that the *in utero* electroporation technique by itself does not affect the development of functional excitatory synaptic inputs to L2/3 pyramidal neurons. To control for off-target effects of Cre recombinase and GFP expression on mEPSC recordings, we also examined mEPSC frequency and amplitude in PirB^+/+^ tissue subsequent to electroporation with GFP.Cre. In this case, Cre recombinase does not cause excision, and thus labeled and unlabeled cells differ only in their expression of GFP and Cre. Results show that GFP^+^;Cre^+^ neurons in PirB^+/+^ slices do not differ significantly from GFP^−^;Cre^−^ neurons in PirB^+/+^ slices in either mEPSC frequency (Fig. 4*K*,*L*) or amplitude (Fig. 4*M*,*N*). This experiment demonstrates that the electroporation and expression of GFP and Cre in and of themselves do not alter detectably these electrophysiological properties of L2/3 neurons. Together, these observations suggest that PirB is required to regulate the functional excitatory synaptic inputs of the neuron, and that the loss of PirB just from the isolated cell examined is sufficient to account for changes in the density of both functional synapses and spines observed in PirB^−/−^ mice.

It should be noted that the 59% increase in mEPSC frequency observed in GFP^+^;Cre^+^ neurons in PirB^fl/fl^ mice (Fig. 4*G*,*H*) is similar to the average 69% increase in spine density on both apical and basal dendrites (Fig. 2*G–J*), implying that many, if not all, of the supernumerary dendritic spines represent sites of functional excitatory synapses.

### mEPSC frequency is not altered in wild-type neighbors located within 100 µm of L2/3 pyramidal neurons lacking PirB

**Figure 5.**
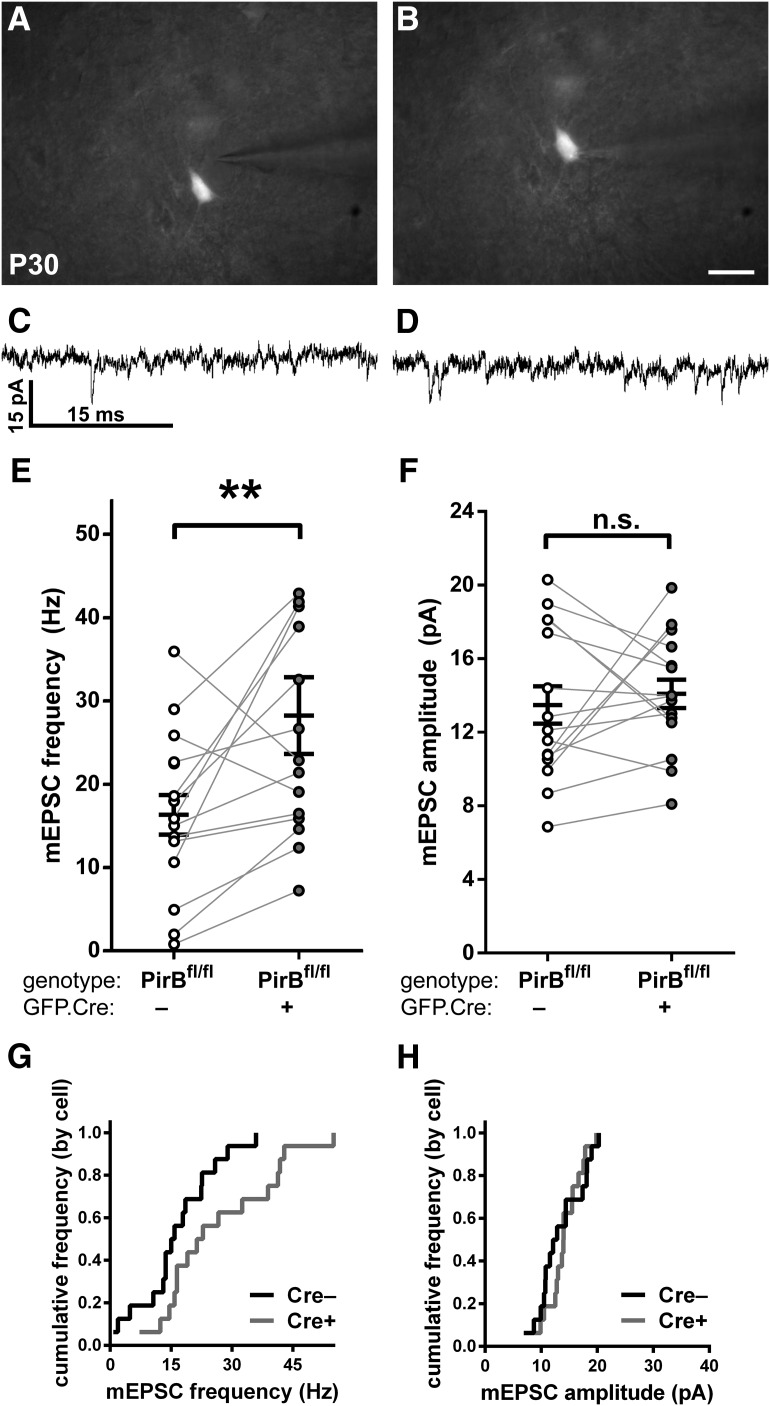
Sparse excision of PirB at E15.5 increases the mEPSC frequency of targeted L2/3 pyramidal neurons in visual cortex, but not in unmanipulated neighboring cells. ***A***, ***B***, Combined differential interference contrast and fluorescence micrograph of visual cortical slice used for whole-cell recordings, showing electrode targeting of a GFP^−^;Cre^−^ cell (***A***) or an isolated GFP^+^;Cre^+^ cell (***B***). ***C***, ***D***, Example traces of mEPSC events in GFP^−^;Cre^−^ (***C***) or GFP^+^;Cre^+^ (***D***) L2/3 pyramidal neurons in slices of visual cortex from P30 PirB^fl/fl^ mice. ***E***, In PirB^fl/fl^ mice, GFP^+^;Cre^+^ cells have higher mEPSC frequency compared with GFP^−^;Cre^−^ cells (GFP^−^;Cre^−^: 16.36 ± 2.38 Hz, *n* = 16 cells, 10 slices, 7 mice; GFP^+^;Cre^+^: 28.26 ± 4.60, *n* = 16 cells, 10 slices, 7 mice; *p* = 0.0060^l^, Wilcoxon signed-rank test). ***F***, mEPSC amplitude is not significantly different in GFP^−^;Cre^−^ vs GFP^+^; Cre^+^ cells (GFP^−^;Cre^−^: 13.49 ± 1.01 pA, *n* = 16 cells, 10 slices, 7 mice; GFP^+^;Cre^+^: 14.10 ± 0.77, *n* = 16 cells, 10 slices, 7 mice; *p* = 0.7722^m^, Wilcoxon signed-rank test). ***G***, ***H***, Cumulative histograms (by cell) of data presented in ***E*** and ***F***, respectively. Scale bars: ***A***, ***B***, 25 μm. **p < 0.01

To test further for a cell-intrinsic effect of PirB on the density of functional synaptic inputs, patch-clamp recordings were made from GFP^+^;Cre^+^ and GFP^−^;Cre^−^ L2/3 pyramidal neurons that were in close proximity, separated by ≤100 μm from each other (Fig. 5). Within this distance, neurons are known to have a much higher connection probability (Perin et al., 2011; Hill et al., 2012; Jouhanneau et al., 2015). In recordings from pairs of neurons located ≤100 μm from each other (Fig. 5*A–D*), we found that mEPSC frequencies in GFP^+^;Cre^+^ neurons were almost always greater than those for the unlabeled GFP^−^;Cre^−^ neighbor (14 of 16 pairs; Fig. 5*E*,*G*). Once again, mEPSC amplitudes between GFP^+^;Cre^+^ and GFP^−^;Cre^−^ neighbors did not differ significantly (Fig. 5*F*,*H*). The average mEPSC frequency of GFP^+^;Cre^+^ neurons (Fig. 5*E*) was similar to that previously recorded from L2/3 pyramidal neurons in germline PirB^−/−^ mice (Djurisic et al., 2013). The mEPSC frequencies of neighboring GFP^−^;Cre^−^ neurons (Fig. 5*E*) were close to previously reported levels in PirB^+/+^ slices (Djurisic et al., 2013).

### Bouton density of axons arising from L2/3 neurons lacking PirB and arborizing in L5 is unaltered

**Figure 6.**
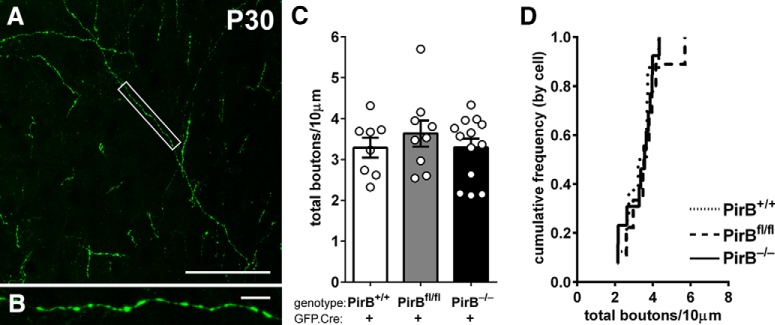
Bouton density on intracortical axons of L2/3 pyramidal cells is not changed with either sparse or germline deletion of PirB. ***A***, Example fluorescence micrograph of boutons along axons within layer 5 arising from L2/3 pyramidal neurons. ***B***, High-magnification, maximum-intensity projection of boxed area in ***A***. ***C***, Bouton density is not different among PirB^+/+^, PirB^fl/fl^, and PirB^−/−^ L2/3 pyramidal neurons electroporated with GFP.Cre (PirB^+/+^: 3.3 ± 0.2, *n* = 8 cells, 3 mice; PirB^fl/fl^: 3.6 ± 0.3, *n* = 9 cells, 5 mice; PirB^−/−^: 3.3 ± 0.2, *n* = 13 cells, 5 mice; PirB^+/+^ vs PirB^−/−^, *p* = 1.000^n^; PirB^+/+^ vs PirB^fl/fl^, *p* = 1.000^n^; PirB^fl/fl^ vs PirB^−/−^, *p* = 1.000^n^; one-way ANOVA with *post hoc* Bonferroni’s multiple comparisons test). ***D***, Cumulative histogram of data (by cell). Scale bars: ***A***, 50 μm; ***B***, 5 μm.

Results from experiments described above imply that PirB acting within a single L2/3 neuron can regulate the spine density of that individual neuron. To test whether PirB can regulate axonal bouton density, we also analyzed at P30 the axon collaterals of L2/3 neurons that descend to cortical L5 in visual cortex (Fig. 6*A*,*B*). Note that this analysis was performed in sections from brains perfused with fixative at P30 following *in utero* electroporation at E15.5, similar to the methods used in Figures 1–3. The varicosities shown at high magnification in Figure 6*B* are typical of en passant synaptic boutons. Bouton density of L2/3 pyramidal cell axons within L5 is not altered in GFP-labeled neurons in germline PirB^−/−^ mice compared with PirB^+/+^ mice (Fig. 6*C*,*D*). Furthermore, no change in bouton density was observed in axons of isolated GFP^+^;Cre^+^ L2/3 neurons in sparsely electroporated PirB^fl/fl^ mice (Fig. 6*C*,*D*). Recall that these single axons arising from neurons lacking PirB arborize in a “sea” of wild-type neurons and glia. Thus, this observation further supports the idea that PirB acts in a cell-intrinsic manner to regulate dendritic spine density in layer 2/3 pyramidal cells, leaving other aspects of neuronal structure intact, including axon bouton density and dendritic branching pattern.

## Discussion

Many factors are now known to regulate aspects of dendritic spine shape, size, and stability, including experience, learning, and environmental enrichment (Holtmaat and Svoboda, 2009; Chen et al., 2014), but just how these external activity-dependent signals are read out into lasting changes is still relatively unclear. Here we have shown that PirB, a receptor whose MHC class I ligands are regulated by neural activity (Corriveau et al., 1998; Huh et al., 2000), acts within individual L2/3 pyramidal neurons in a cell-intrinsic manner to regulate spine density and functional excitatory synapses. We have used the powerful technique of *in utero* electroporation of Cre.GFP to target PirB deletion selectively to L2/3 pyramidal neurons. It is remarkable that simply driving PirB excision in single isolated neurons surrounded by wild-type glia and wild-type neurons results in a major increase in spine density within the targeted neuron. This observation indicates that PirB function in individual L2/3 pyramidal neurons is required to regulate spine density. It is also consistent with the previous finding that spine density on L5 pyramidal cells is also elevated in mice lacking PirB (Djurisic et al., 2013). Together, these observations imply that PirB may regulate spine density more generally in excitatory neurons of the forebrain.

At P30, spine density on L2/3 pyramidal neurons lacking PirB is >50% greater than in PirB^+/+^ neurons (Figs. 1, 2). However, just 1 week earlier, at P23, we found that spine density across all genotypes exposed to GFP.Cre at E15.5 is similar, ∼13 spines/10 μm (Fig. 3). Between P23 and P30, spine density on L2/3 PirB^+/+^ neurons falls to about seven spines/10 μm, which is consistent with the idea that this is a peak period for spine and synapse pruning (Ruiz-Marcos and Valverde, 1969; Markus and Petit, 1987). In contrast, spine density on neurons lacking PirB fails to decline and instead remains close to the level at P23. Overall, results strongly suggest that PirB function is required for spine pruning during this period. Given the strong evidence of a role for microglia in spine and synapse pruning (Schafer et al., 2012; Parkhurst et al., 2013), our results also imply that, without neuronal PirB expression, glia may not be able to function properly to eliminate spines.

Several lines of evidence presented here argue that the striking increases in spine density and functional synapses are not artifacts of Cre recombinase or GFP expression. First, a significant increase in spine density is observed when Cre.GFP is electroporated into PirB^−/−^ mice (Fig. 1*E–J*). Second, a similar result is obtained when Cre.GFP is electroporated into PirB^fl/fl^ mice (Fig. 2*D–J*). Third, the independent technique of mEPSC recordings revealed a parallel increase in functional excitatory inputs (Figs. 4, 5), as signaled by the significantly greater mEPSC frequency in PirB^−/−^ versus PirB^+/+^ neurons. Moreover, whole-cell recordings of mEPSCs made from isolated neurons electroporated with Cre.GFP (Figs. 4, 5) are indistinguishable from those recorded in germline PirB^−/−^ neurons (Djurisic et al., 2013). Finally, when Cre.GFP is electroporated into PirB^+/+^ mice, mEPSCs recorded from L2/3 neurons (Fig. 4*K–N*) are similar to those seen in unmanipulated PirB^+/+^ mice not undergoing electroporation (Djurisic et al., 2013).

Here we have uncovered a cell-intrinsic mechanism for maintaining spine density and excitatory synaptic input below an upper bound. L2/3 neurons lacking PirB have as much as a 73% greater spine density than PirB^+/+^ neurons (Figs. 1, 2). The corresponding increase in mEPSC frequency (Fig. 4) suggests that many of the spines on isolated PirB^−/−^ cells are functional, a finding similar to what has been observed on the L5 pyramidal neurons in PirB^−/−^ mice (Djurisic et al., 2013). Moreover, we observed that neurons with intact PirB alleles have lower mEPSC frequencies than their isolated neighbors lacking PirB (Fig. 5). This observation is one of the strongest arguments for a cell-autonomous role for PirB in L2/3 pyramidal neurons. The fact that mEPSC frequency in wild-type neighbors is indistinguishable from that recorded in L2/3 PirB**^+/+^** mice, and that mEPSC frequency in isolated PirB^−/−^ cells is indistinguishable from that recorded in L2/3 neurons in PirB^−/−^ mice, is additional support for this conclusion.

The term “cell autonomous” is used here explicitly in reference to a role for PirB in regulating spine density on L2/3 pyramidal neurons. There are several other examples in which spine density is thought to be regulated by cell-autonomous mechanisms, including NgR1 in the cortex (Akbik et al., 2013) and Sema5A in the hippocampus (Duan et al., 2014). In all of these cases, including PirB, the conclusion regarding cell-autonomous function could not have been achieved without mosaic analysis *in vivo* or *in vitro*. PirB may also have non-cell-autonomous functions. For example, the increase in mEPSC frequency recorded in isolated neurons lacking PirB implies that there has been a parallel increase in the number of presynaptic boutons, which likely derive from a vast majority of PirB^+/+^ neurons. Thus, the spine density increase appears to drive a transynaptic increase in functional inputs: a non-cell-autonomous effect. This interpretation is also consistent with the finding here that in the sparse electroporation experiments, the axon bouton density of neurons lacking PirB does not differ from that of PirB^+/+^ neurons (Fig. 6). These mutant axons are embedded in a sea of PirB^+/+^ neurons possessing normal spine density. A non-cell-autonomous role for PirB in regulating presynaptic boutons would predict that the density of axonal inputs belonging to PirB^−/−^ neurons should be wild type, exactly what we have observed.

There is a growing list of molecules known to regulate spines and excitatory synaptic inputs to cerebral cortical pyramidal cells. For instance, FMRP regulates spine density and maturation; in the knockout, spine density is increased, but spines remain thin and immature, and can be rescued by reducing mGluR5 expression (Dölen et al., 2007). Another receptor, DR6, regulates the density of axonal boutons and sprouting following activity-dependent deprivation, but there is no known effect on the normal developmental spine-pruning process (Marik et al., 2013). NgR1 is thought to regulate the density of mature dendritic spines (Karlsson et al., 2016); however, there is currently some disagreement about its exact role in dendritic spine and axonal bouton turnover (Akbik et al., 2013; Park et al., 2014; Frantz et al., 2016). Many downstream effectors have been identified and studied, including Rho GTPases (Murakoshi et al., 2011; Colgan and Yasuda, 2014) and the actin cytoskeleton (Kim et al., 2013; Kellner et al., 2016). In contrast, our studies suggest that PirB acts to keep spine density below a ceiling level (Djurisic et al. 2013), with no apparent effect either on the distribution of spine types (Bochner et al., 2014) or on the overall dendritic morphology (Figs. 1*K*,*L*, 2*K*,*L* ). Spine motility on L5 pyramidal neurons lacking PirB is also decreased (Djurisic et al., 2013), implying a connection between PirB and downstream signaling to cofilin and the actin cytoskeleton (Kim et al., 2013). Clearly, every aspect of the spine is tightly regulated to enable experience-dependent changes to be encoded structurally. Together, the results of our studies suggest that PirB is needed to match spine density and excitatory synaptic function to activity levels within cortical circuits, thereby providing headroom for the cell to encode additional experiences at new synapses.

## Synthesis

Reviewing Editor: Lorna Role, Stony Brook <br /> Decisions are customarily a result of the Reviewing Editor and the peer reviewers coming together and discussing their recommendations until a consensus is reached. When revisions are invited, a fact-based synthesis statement explaining their decision and outlining what is needed to prepare a revision will be listed below. The following reviewer(s) agreed to reveal their identity: Julie Lefebvre.

In eN-NWR-089-16 the investigators tested whether paired immunoglobulin receptor B (PirB) plays a role in determining the number of dendritic spines and the extent of spontaneous excitatory synaptic input to Layer II/III pyramidal neurons in the visual cortex. The authors use techniques that allowed for sparse neuronal deletion of PirB and examined the effects on individual PirB-/- neurons "in a sea" of PirB +/+. Specifically, they used timed in utero electroporation to co-express GFP and Cre recombinase in a laminar- and cell type-specific manner, and to disrupt PirB expression in very few layer II/III pyramidal neurons following Cre-mediated excision of floxed PirB alleles.

Single cell analysis reveals higher spine density and increased mEPSP frequency in individual pyramidal neurons as a result of sparse Cre-mediated PirB deletion. In contrast, loss of PirB expression (by either germline or sparse deletion) does not appear to affect dendritic branching complexity or axonal bouton density as determined by examination of Layer II/III axons projecting within Layer V.

These findings support the idea that PirB regulation of Layer II/III pyramidal neurons occurs in a cell-autonomous manner to maintain normal density of dendritic spines and functional excitatory synapses. The authors further suggest that under normal conditions PirB might provide an important, cell autonomous, limitation on the number of excitatory synapses on Layer II/ III pyramidal neuron.

The presentation of the data are generally clear and compelling (and the figures are beautiful). However, the reviewers had some additional questions that should be addressed to improve the accessibility and impact of the manuscript.

Major Issues to address

1.Both reviewers asked for more explication of the specific PirB -/- and PirB fl/fl lines used and the specific nature of the resultant mutations in both the germline and sparse deletion conditions:

"The information provided for the mouse lines used in the study is very limited. It is not clear if genetic targeting in PirB-/- mice (and also Cre-mediated excision in PirB fl/fl mice) results in null deletion or truncation of PirB.

"In Syken et al, only one mouse line was described and called PirBTM; it creates a truncation and [ it is] unable to signal. It is not clear if this refers to PirB-/-. Also Citation is needed for PirB f/f (Bochner et al 2014). "

2. Both reviewers agreed that inclusion of additional validation related data would be helpful ...

"The manuscript should include the results from validation studies of the GFP.Cre construct. For example, % of GFP positive cells tested immunonegative for PirB."

"The degree of transfection by IUE can vary. Can the authors comments on the variability, and how samples were chosen for analysis? It seems to me that a large density of transfected cells could still present a non-autonomous effect through coupling. Though recordings in Fig 3 aim to address this, were cells chosen in a relatively sparse or dense transfected region? Do the authors have examples of spine density counts in cells in sparsely transfected samples?"

3. Both reviewers felt that the issue of strict neuronal autonomy was not clear, especially in the context of specific comments by the authors in text at lines 350 - 353. Likewise, both reviewers felt that discussion of potential mechanisms (pruning etc) was not addressed experimentally and as such authors cannot fairly discuss neuronal PirB function vis-a-vis the role of microglia-dependent pruning.

"In the [electroporation] studies, how does spine density in PirB-/- compare to PirB f/f mutants? This is presented separately in Figs 1 and 4, but a statistical comparison and discussion of the mutant defects for both genotypes (appear to be similar) would be valuable here."

"Comparison of spine density in PirB -/- vs PirB fl/fl should.. address.. whether the contribution is solely cell-autonomous, of whether there are additional contributions from other cells. If it is the latter, than the density in PirB-/- would be greater than in single PirB f/f neurons; but [the] data on average spine density [shown] in Fig 1 (PirB-/-) vs Fig 4 (PirB f/f) appear to be similar.This analysis could be further discussed to emphasize the **cell autonomous **role for neuronal PirB in limiting spine density.

"Lines 350 - 353: This sentence describing possible involvement of microglia does not make logical sense; it is in contradiction with the cell-autonomous function of neuronal PirB. "

"This study doesn't address whether synaptic pruning is the mechanism, and perhaps this should be pushed to a later section of the discussion"

4. Both reviewers felt that additional discussion/ explication of the axonal changes (or lack thereof) would be of interest.

"PirB is expressed in cortical pyramidal neurons. Although loss of PirB expression in pyramidal neurons leads to increased spine density, it has no apparent effects on axonal bouton density. A discussion of differential effects of PirB on dendritic spines and axonal boutons in the context of normal neuronal PirB function would be of likely interest to the readers."

Other points:

1. There were some issues identified pertinent to the statistical data presented and analyses used as well as the presentation for the Sholl analyses.

- both reviewers asked for the n values to be listed both in terms of numbers of animals as well as number of cells.

- one reviewer commented that the Kolmogorov-Smirnov test is a preferred post hoc test for comparison of cumulative distributions (rather than the two-way ANOVA and Bonferroni's post hoc test on multiple comparisons that was used.

- one reviewer commented that the current display of the Sholl data in continuous lines x distance was suboptimal "It is not clear why the data obtained at distance intervals are presented as line graphs with connecting data points." Whereas the other review felt that either "line plots, or bar graphs plotted for each distance measured" was acceptable. We leave this decision to the authors, but refer the authors to the presentation in Metz AE, Yau HJ, Centeno MV, Apkarian AV, Martina M (2009) Morphological and functional reorganization of rat medial prefrontal cortex in neuropathic pain. Proc Natl Acad Sci USA 106(7):2423-8).

2. Both reviewers thought it would be helpful if there were additions to Figures 1 and 4-specifically, to include high-resolution images showing dendritic spines along apical and basal dendrites of layer II/III pyramidal neurons - this would better illustrate increased spine density on the GFP+/Cre+ neurons of PirB-/- and PirBfl/fl mice.

3. Both reviewers asked that the authors indicate that the values from GFP+/Cre+ neurons in PirB +/+ mice in Fig 1E-F are used again as the control values in Fig 4A-B.

4. There was also concern about the appearance of the axon segment shown in Figure 5.. The observed blebbing is typically a sign of poor health due to fluorescence illumination. Can the authors comment why they identify these en passant swellings as normal release sites, please.

Minor points to address:

1. Several references are missing from the list. For example, Hayashi-Takagi et al., 2015.

2. Line 57: It should be "Frantz et al., 2016" not "Frantz et al., 2015".

3. Line 141: What is the resolution of high-resolution images acquired in the present study?

4. Line 202: Why are these two references cited for analysis parameters?

5. Lines 243 - 246: This sentence does not make logical sense.

6. Lines 254 - 256: In this concluding statement, the authors also refer to results from the other study. This is very confusing to the readers.

7. Figures 1 and 2: Images captured at higher magnification are shown without indication of their origin in the low-magnification images (e.g., Fig 1C in 1A, Fig 1D in 1C and Fig 2C in 2B).

8. Line 265: "Cells expressing GFP indicate Cre-driven excision of the floxed PirB alleles." This sentence makes the paragraph (lines 276 - 290) very confusing as there are labeled (GFP+; Cre+) neurons in both sets of experiments, i.e., Fig 2H-K and Fig 2L-O. Text should be revised to make the distinction (i.e., Cre-mediated excision of floxed PirB in Fig 2H-K vs. no excision of PirB in Fig 2L-O) clear to the readers.

9. Lines 287 - 290: See point #6.

10. Figures 3G and 3H: These two figures are not mentioned in the text.

11. Line 301: The authors need to provide % increase in mEPSC frequency (Fig 3E) because comparisons are later made in lines 315 - 317.

12. The authors indicate in figure legends the number of neurons that were analyzed. In each figure, it should be indicated how many neurons were analyzed, from how many animals.

13. Neurons are referred to as "Cre+" and "Cre-" in figure legends but they are referred to as "GFP+; Cre+" and "GFP+; Cre-" in the text. The naming should be consistent.

14. Line 653: Is it "(by slice)" or "(by cell)"?
